# Placental malaria is associated with attenuated CD4 T-cell responses to tuberculin PPD 12 months after BCG vaccination

**DOI:** 10.1186/1471-2334-12-6

**Published:** 2012-01-14

**Authors:** Brigitte Walther, David JC Miles, Pauline Waight, Melba S Palmero, Olubukola Ojuola, Ebrima S Touray, Hilton Whittle, Marianne van der Sande, Sarah Crozier, Katie L Flanagan

**Affiliations:** 1 MRC Laboratories Gambia, PO Box 273, Banjul, Gambia; 2 Malawi-Liverpool-Wellcome Trust Clinical Research Programme, PO Box 30096, Chichiri, Blantyre 3, Malawi; 3 Immunisation Department, Health Protection Agency Centre for Infections, London, UK; 4 Department of Pediatrics, Bronx Lebanon Hospital Center, 1650 Selwyn Avenue, Bronx, New York, USA; 5 Epidemiology and Surveillance Unit, Centre for Infectious Diseases Control, National Institute for Public Health and the Environment, Bilthoven, The Netherlands; 6 Julius Centre, University Medical Centre Utrecht, Utrecht, The Netherlands; 7 MRC Epidemiology Resource Centre, University of Southampton, Southampton General Hospital, Southampton, UK

## Abstract

**Background:**

Placental malaria (PM) is associated with prenatal malaise, but many PM+ infants are born without symptoms. As malaria has powerful immunomodulatory effects, we tested the hypothesis that PM predicts reduced T-cell responses to vaccine challenge.

**Methods:**

We recruited healthy PM+ and PM- infants at birth. At six and 12 months, we stimulated PBMCs with tuberculin purified protein derivative (PPD) and compared expression of CD154, IL-2 and IFNγ by CD4 T-cells to a negative control using flow cytometry.

We measured the length, weight and head circumference at birth and 12 months.

**Results:**

IL-2 and CD154 expression were low in both groups at both timepoints, without discernable differences. Expression of IFNγ was similarly low at 6 months but by 12 months, the median response was higher in PM- than PM + infants (*p *= 0.026). The PM+ infants also had a lower weight (*p *= 0.032) and head circumference (*p *= 0.041) at 12 months, indicating lower growth rates.

At birth, the size and weight of the PM+ and PM- infants were equivalent. By 12 months, the PM+ infants had a lower weight and head circumference than the PM- infants.

**Conclusions:**

Placental malaria was associated with reduced immune responses 12 months after immune challenge in infants apparently healthy at birth.

## Background

*Plasmodium falciparum *malaria is endemic in much of Sub-Saharan Africa and commonly infects the placentas of pregnant women. The overt consequences of placental malaria (PM) include high risk of premature birth and low birth weight [1,2], increased neonatal mortality [3] and infant anaemia [4]. Exposure to PM has been associated with an increased risk for malaria during the first years of life [5].

Placental malaria has been associated with poor cytokine production by T-cells [6,7] and induced tolerance to *Plasmodium *antigens [8], which may be associated with the induction of regulatory T-cells in PM-exposed infants [9-11]. These findings are consistent with earlier results that showed that malaria in children led to reduced antibody responses to vaccination with bacterial polysaccharide, glycoconjugate and protein antigens [12-16], and also that prenatal exposure to malaria is associated with relatively low cytokine responses to *P. falciparum *antigens for at least the first three years of life [8].

The effect of malaria may be partly responsible for the observation that in regions of Sub-Saharan Africa where *P. falciparum *malaria is endemic, several vaccines used for routine childhood immunisation are frequently less effective than in high income countries, which was the subject of a recent review [17].

One of the best documented examples is that of the Bacille Calmette-Guérin (BCG) vaccine for tuberculosis, which has demonstrated good protection in most trials carried out in high income countries but little or no protection in trials carried out in low income tropical countries [18]. Mechanisms of protection against tuberculosis have not been defined, but defects in IFNγ signalling are strongly predictive of disease so it is likely that the T-cell response plays an important part in protection [19-21].

A possible explanation for the low levels of protection afforded by BCG in sub-Saharan Africa is offered by the finding that BCG does not induce the same level of T-cell IFNγ production in Malawian children as it does in British children [22,23].

We hypothesised that the immunomodulatory effects of *P. falciparum *infection of the placenta extend to immune challenges encountered during the early postnatal period. We conducted a preliminary study using an existing dataset derived from the Sukuta Birth Cohort in The Gambia, which was collected in order to study the development of CD4 T-cell responses to cytomegalovirus (CMV) in early life [24,25], to establish whether there was an association between PM and CD4 T-cell responses to the BCG vaccination.

## Methods

### Cohort characteristics

Healthy infants were recruited from the maternity ward of Sukuta Health Centre, following written informed consent from both parents. Sukuta Health Centre serves a low income, peri-urban community in The Gambia. Malaria transmission rates are at their highest during and immediately after the wet season, giving rise to a distinct malaria season from August to December [25]. Recruitment took place from January to August, 2002.

Infants were recruited at birth, and the cohort was restricted to healthy infants by excluding infants with a birthweight below 2.0 kg, infants born from pregnancies with complications that required hospital admission and infants with congenital abnormalities. Twins were also excluded.

From the existing dataset, we identified a PM+ group consisting of all infants exposed to placental malaria whose response to PPD had been measured at either six (23-30 weeks) or 12 months (46-58 weeks) or both. We then identified a PM- group of all infants whose response to PPD had been measured at the same ages, and whose placentas showed no signs of malarial infection.

At birth, the APGAR score at 5 min was recorded. Babies were weighed, the length was measured with a measuring board, and a tape measure was used to measure the head and mid-upper arm circumferences. The weight, length and head circumference were measured again at 12 months.

All infants were immunised intradermally with BCG within 24 h of birth according to the Gambian expanded program of immunisation.

Mothers were requested to bring infants to the clinic if they became ill at any point during follow up, where treatment was provided and details of the illnesses recorded.

The study was approved by the Gambia Government/MRC Laboratories Joint Ethics Committee.

### Intracytoplasmic cytokine staining

**Figure 1:**
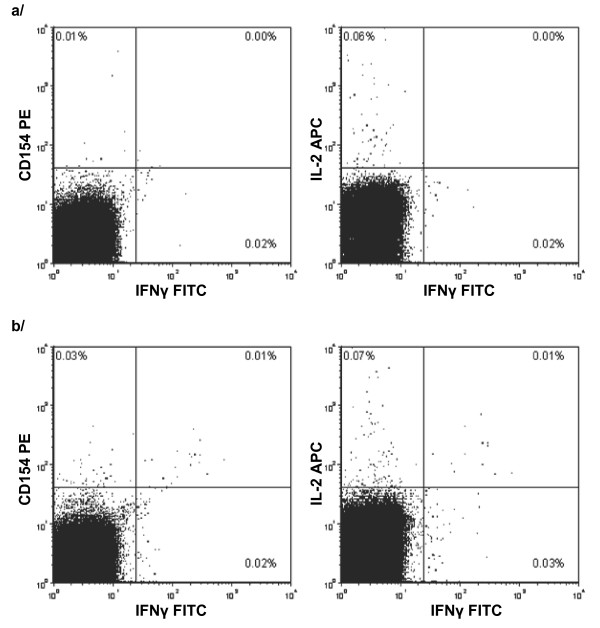
**Gating of CD4 T-cells expressing IFNγ, IL-2 and CD154**. Following selection of lymphocytes by forward and side scatter characteristics and CD4 T-cells by gating on high expression of CD4, quadrant gates were set on a/the negative control sample and then applied to b/the PPD-stimulated sample. Sample selected as representative as it has the median CD154 response among PM- infants at 12 months.

The PBMCs were isolated by density gradient centrifugation using lymphoprep (Axis-Shield POC AS, Oslo, Norway) and resuspended at 2 × 10^6 ^cells ml^-1 ^in R10F (90% v/v RPMI-1640 containing 100 U ml^-1 ^penicillin plus 100 μg ml^-1 ^streptomycin, 10% v/v fetal bovine serum) and treated with either 10 μg ml^-1 ^of the RT49 preparation of tuberculin purified protein derivative (PPD) (Statens Serum Institut, Copenhagen, Denmark), 10 μg ml^-1 ^lysed CMV-infected human dermal fibroblast cells (Virusys, Taneytown, MD, USA), or 10 μg ml^-1 ^normal human dermal fibroblast lysate (NHDF) (Virusys) [26] as a negative control, as required by the CMV study [24]. After 2 h at 37°C in a 5% carbon dioxide atmosphere, cytokine secretion was inhibited with 10 μg ml^-1 ^brefeldin A (Sigma, Natick, MA, USA) and the PBMCs were incubated for a further 16 h. The PBMCs were concentrated by centrifugation, permeabilised using FACSperm II solution (BD Biosciences, Franklin Lakes, NJ, USA), and stained with PerCP-conjugated anti-CD4 antibodies, FITC-conjugated anti-IFN-γ antibodies, PE-conjugated anti-CD154 antibodies, and APC-conjugated anti-IL-2 antibodies. All antibodies were obtained from BD. The stained cells were stored in 2% v/v formalin in phosphate-buffered saline at 4°C and as many cells as possible were acquired on a four-color FACScalibur (BD), and analyzed using FCS Express (De Novo Software, Los Angeles, CA, USA). CD4 T-cells were identified as having high levels of staining for CD4 (median number of CD4 T-cells 28,506, IQR 9,899-61,453). Cells expressing markers of interest were identified by setting gates on negative control cells to identify non-responding cells, and applying the same gates to the PPD-stimulated cells (Figure 1). The PPD and CMV-specific responses were defined as the percentage of CD4 T-cells expressing each marker among the PPD-treated cells after subtraction of the percentage of CD4 T-cells expressing each marker among the negative control cells.

### Diagnosis of placental malaria

PM was diagnosed from a 1 cm × 2 cm × 2 cm biopsy of the placenta that was stained with haematoxylin and eosin and examined under a light microscope by two trained diagnosticians [10]. If evidence of malaria infection was found, it was classified as past, acute or chronic infection [27].

### Statistical analysis

Data on PPD responses were available from 24 infants at 6 months, of whom five were PM+ at birth. Data were available from 35 infants at 12 months, of whom seven were PM+ at birth. The low numbers made it impossible to analyse acute, chronic and past infection separately, so infants with any presentation of PM were classified as PM+ and compared with PM- infants.

Mann-Whitney U tests were used to compare measurements of weight, size, APGAR score and CD4 responses to PPD. Statistical analysis were performed with Stata 9.1 (StataCorp LP, College Station, TX, USA) and MINITAB 14 (Minitab inc, State College, PA, USA).

## Results

### Clinical characteristics

The size, weight and APGAR scores of PM+ and PM- infants were equivalent at birth (Table 1). By 12 months of age, the median weight and head circumference of the PM- infants were greater than those of the PM+ infants, although there was no discernable difference in length (Table 2).

**Table 1:** Size, weight and APGAR scores of PM+ and PM- infants at birth

	PM+ (n = 7)		PM- (n = 28)	
Parameter	**Median**	**IQR**	**Median**	**IQR**

APGAR score	9	9-10	9	9-10

Birthweight (kg)	2.95	2.73-3.11	2.93	2.70-3.40

Length (cm)	49	48-51	49	48-50

Head circumference (cm)	34	33-34	34	33-35

Mid-upper arm circumference (cm)	10.6	9.7-11.0	9.8	9.6-10.5

None of the differences were significant

**Table 2:** Weight, length and head circumference measured at 12 months of age

	PM+ (n = 7)		PM- (n = 28)		*p*
Parameter	**Median**	**IQR**	**Median**	**IQR**	

Weight (kg)	8	7.30-8.46	9.14	8.05-10.18	0.032

Length (cm)	72.8	71.0-74.4	73.5	72.5-75.4	NS

Head circumference (cm)	44.5	43.5-45.0	45.5	44.6-46.38	0.041

Comparisons by Mann-Whitney U test. NS indicates non-significant

Of the five PM+ infants sampled at six months, two were diagnosed with acute infection, one with chronic infection and two with past infection. Of the seven PM+ infants sampled at 12 months, three had acute infection, one had chronic infection and three had past infection.

During the follow-up period, malaria infections were diagnosed in three of the 28 PM- infants and none of the seven PM+ infants, which was not a significant difference.

### Less CD4 T-cells produced IFNγ in response to PPD following exposure to placental malaria

**Figure 2:**
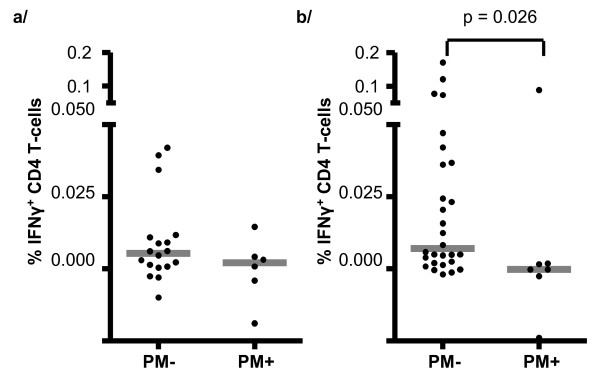
**Percentages of CD4 T-cells that expressed IFNγ at a/6 months and b/12 months compared by placental malaria (PM) status of the infant**. Bars indicate medians. Statistical comparisons by Mann-Whitney U test.

At 6 months, very few PPD-specific IFNγ-producing CD4 T-cells were found in any of the infants, and there were no significant differences in responses between PM+ (median 0.002%, IQR -0.008; 0.007%) and PM- (median 0.005%, IQR 0.001; 0.011%) groups. By 12 months of age, most of the PM- infants had at least some PPD-specific response, and the proportion of PPD-specific IFNγ-producing CD4 T-cells was significantly higher in PM- (median 0.007%, IQR 0.002; 0.037) than PM+ infants (median 0.000%, IQR -0.002; 0.002%) (*p *= 0.026) (Figure 2).

### Few CD4 T-cells responded to PPD by producing IL-2 or CD154

The median PPD-specific IL-2 and CD154 responses of both PM+ and PM- infants were negative at 6 months, indicating that percentages of responding PPD-treated cells were below negative control levels. By 12 months the median PPD-specific response of PM- infants was positive, albeit very low, for IL-2 (median 0.002%, IQR -0.002; 0.009) and CD154 (median 0.003%, IQR -0.026; 0.009). Median responses of PM+ infants remained negative and very close to zero, but there was no significant difference between PM+ and PM- groups.

### No association between PM and responses to CMV

As the majority of the infants were infected with CMV, we also compared the CD4 T-cell responses to CMV lysate between PM- and PM+ infants. The numbers available were 5 PM+ and 20 PM- at 6 months, and 5 PM+ and 24 PM- at 12 months.

We found no significant differences in the percentages of CD4 T-cells expressing IFNγ, IL-2 or CD154 between PM+ and PM- infants, either at six or 12 months of age (data not shown).

## Discussion

Our principal finding is that placental malaria predicted a relatively weak IFNγ response to PPD a year after birth, which was also a year after the administration of the BCG vaccine. No difference was detectable at 6 months, probably because of the low response in both groups.

By contrast, we did not find an association between PM and responses to CMV. There are distinct differences in the presentation of BCG and CMV, as the BCG vaccine is administered within 24 h of birth and is present for a limited period of time. Gambian infants are usually infected with CMV several weeks after birth [28], after which it establishes a persistent infection so that the host is exposed to CMV antigens for the rest of their life [29]. The contrast between the associations with BCG and CMV suggests two mutually compatible explanations. The first is that the influence of PM on the response to BCG is stronger because the exposure to BCG occurred chronologically closer to the exposure to the exposure to placental malaria. The other is that the persistently high antigen exposure in CMV-infected infants drove the immune response to overcome any deleterious influence of PM.

The lack of effect on responses to CMV contrasts with earlier findings that malaria episodes are associated with low responses to another persistent herpesvirus, Epstein-Barr virus (EBV), and also with higher EBV viral loads [30-32], implying poor immune control of viral replication. However, our study differs substantially from the studies that generated these findings as apart from the fact that the natural histories of CMV and EBV infection are very different, the children in our study were younger than most of those in the previous studies. Further, the previous studies were on children who either had a recent malaria episode or were frequently infected, while the infants in our cohort were exposed to PM and very few developed malaria in the subsequent year, implying little or no exposure to *P. falciparum*.

We have previously reported that in BCG-vaccinated infants below 12 months, few CD4 T-cells produce IFNγ in response to PPD [33], so it is likely that differences associated with PM were simply undetectable until the CD4 T-cells of the infants were producing enough IFNγ for a difference to be apparent.

The numbers were too small to analyse for the effects of acute, chronic and past PM. However, the relative proportions of the three types were similar in the infants sampled at six and 12 months, making it unlikely that the appearance of a difference at 12 months was due to a difference in the type of PM among the PM+ infants.

The sizes and weights of infants at birth in the PM+ and PM- groups were comparable, so we could exclude the confounding effects of premature birth and low birth weight which are well described for PM [1,2]. The exclusion criterion of low birthweight also ruled out the effect of prenatal undernutrition, which is associated with poor immune development [34,35] and while we have previously found an association between PM and congenital cytomegalovirus infection [36], we found no evidence that congenital cytomegalovirus modulated the responses of CD4 T-cells to PPD [24] in a much larger and more powerful study.

Testing for HIV was not carried out, but a survey carried out in the nearby town of Serrekunda from 2000 to 2001 found a prevalence of 1.0% among pregnant women [37], and a recent screen of women in Sukuta confirmed the rates to be < 1% (unpublished), thus HIV was unlikely to be a serious confounder.

Helminth infection has been shown to modulate immune responses to mycobacterial antigens in BCG-vaccinated children [38-40], but we found that intestinal helminth infections are not endemic in infants in the Sukuta area after a screen of fecal samples collected at 12 months of age (unpublished). However, the possibility has been raised that helminth infection during pregnancy may reduce the IFNγ response to PPD [41], which we cannot eliminate as we did not screen the mothers for intestinal infections.

By 12 months of age, the PM+ infants were slightly smaller than the PM- infants. As there were no differences at birth, the difference at 12 months implies that PM was associated with reduced growth, which concurs with our findings from the entire cohort from which infants were selected for the analysis presented here [25]. Similarly, the differences in IFNγ response emerged between 6 and 12 months, suggesting that PM was predictive of deleterious effects that remained present 12 months after birth.

Although no correlate of protection to tuberculosis has been identified, the ability to generate IFNγ-producing T-cells has been shown to be important, as defects in IFNγ signalling lead to a high susceptibility to tuberculosis disease [19-21]. While it is possible that the reduced number of IFNγ-producing cells indicated a shift in response profile, rather than an overall loss of T-cell response, it is still likely that a reduced number of IFNγ-producing CD4 T-cells represents a reduced level of protection.

While we detected an association between PM and reduced immune response to BCG vaccination at birth, the scope of the study did not extend to establishing whether there was a direct causal link. Although PM makes infants more susceptible to subclinical *P. falciparum *infection and episodes of malaria [5,8,42], it is unlikely that the lower responses of PM+ infants were due to the immunosuppressive effect of repeated malaria episodes [43,44] as we did not find a higher rate of malarial disease in PM + infants. While we could not exclude all possible confounders, no effect that we were able to measure showed any association with reduced responses other than exposure to PM.

## Conclusions

The fact that an association between PM and reduced immune responses was detectable with such low numbers suggests a strong association between PM and reduced CD4 T-cell response. As subclinical PM is common in Sub-Saharan Africa, a study on a large enough scale to confirm our findings and investigate the mechanism may yield results that are important for infant health in the region.

## Competing interests

The authors declare that they have no competing interests.

## Authors' contributions

BW, DJCM, MvdS and HW conceived and designed the study. DJCM, MvdS, OO, EST, KLF and MSP implemented the study and collected the data. BW, DJCM, PW and SC interpreted and analysed the data. DJCM and BW drafted the manuscript. All authors critically reviewed and approved the final manuscript.

## Pre-publication history

The pre-publication history for this paper can be accessed here:

http://www.biomedcentral.com/1471-2334/12/6/prepub

## References

[B1] Sullivan A Nyirenda T Cullinan T Taylor T Harlow S James S Meshnick S Malaria infection during pregnancy: intrauterine growth retardation and preterm delivery in Malawi J Infect Dis 1999 179 6 1580 1583 10.1086/314752 10228088

[B2] Luxemburger C McGready R Kham A Morison L Cho T Chongsuphajaisiddhi T White N Nosten F Effects of malaria during pregnancy on infant mortality in an area of low malaria transmission Am J Epidemiol 2001 154 5 459 465 10.1093/aje/154.5.459 11532788

[B3] Garner P Gülmezoglu A Drugs for preventing malaria-related illness in pregnant women and death in the newborn Cochrane Database Syst Rev 2003 1 CD000169 10.1002/14651858.CD000169 12535391

[B4] Le Cessie S Verhoeff F Mengistie G Kazembe P Broadhead R Brabin B Changes in haemoglobin levels in infants in Malawi: effect of low birth weight and fetal anaemia Arch Dis Child Fetal Neonatal Ed 2002 86 3 F182 F187 10.1136/fn.86.3.F182 11978749 PMC1721412

[B5] Le Hesran J Cot M Personne P Fievet N Dubois B Beyemé M Boudin C Deloron P Maternal placental infection with *Plasmodium falciparum *and malaria morbidity during the first 2 years of life Am J Epidemiol 1997 146 10 826 831 9384203 10.1093/oxfordjournals.aje.a009200

[B6] Broen K Brustoski K Engelmann I Luty A Placental *Plasmodium falciparum *infection: causes and consequences of in utero sensitization to parasite antigens Mol Biochem Parasitol 2007 151 1 1 8 10.1016/j.molbiopara.2006.10.001 17081634

[B7] Ismaili J van der Sande M Holland M Sambou I Keita S Allsopp C Ota M McAdam K Pinder M *Plasmodium falciparum *infection of the placenta affects newborn immune responses Clin Exp Immunol 2003 133 3 414 421 10.1046/j.1365-2249.2003.02243.x 12930369 PMC1808798

[B8] Malhotra I Dent A Mungai P Wamachi A Ouma J Narum D Muchiri E Tisch D King C Can prenatal malaria exposure produce an immune tolerant phenotype? A prospective birth cohort study in Kenya PLoS Med 2009 6 7 e1000116 10.1371/journal.pmed.1000116 19636353 PMC2707618

[B9] Bisseye C van der Sande M Morgan W Holder A Pinder M Ismaili J *Plasmodium falciparum *infection of the placenta impacts on the T helper type 1 (Th1)/Th2 balance of neonatal T cells through CD4^+^CD25^+ ^forkhead box P3^+ ^regulatory T cells and interleukin-10 Clin Exp Immunol 2009 158 3 287 293 10.1111/j.1365-2249.2009.04014.x 19758375 PMC2792824

[B10] Flanagan K Halliday A Burl S Landgraf K Jagne Y Noho-Konteh F Townend J Miles D van der Sande M Whittle H The effect of placental malaria infection on cord blood and maternal immunoregulatory responses at birth Eur J Immunol 2010 40 4 1 11 10.1002/eji.200939638 20039298

[B11] Brustoski K Möller U Kramer M Hartgers F Kremsner P Krzych U Luty A Reduced cord blood immune effector-cell responsiveness mediated by CD4^+ ^cells induced in utero as a consequence of placental *Plasmodium falciparum *infection J Infect Dis 2006 193 1 146 154 10.1086/498578 16323143

[B12] McGregor I Barr M Antibody response to tetanus toxoid inoculation in malarious and non-malarious Gambian children Trans R Soc Trop Med Hyg 1962 56 5 364 367 10.1016/0035-9203(62)90005-6

[B13] Greenwood B Bradley-Moore A Bryceson A Palit A Immunosuppression in children with malaria Lancet 1972 299 7743 169 172 10.1016/S0140-6736(72)90569-7 4109547

[B14] Williamson W Greenwood B Impairment of the immune response to vaccination after acute malaria Lancet 1978 311 8078 1328 1329 10.1016/S0140-6736(78)92403-0 78096

[B15] Bradley-Moore A Greenwood B Bradley A Bartlett A Bidwell D Voller A Craske J Kirkwood B Gilles H Malaria chemoprophylaxis with chloroquine in young Nigerian children. II. Effect on the immune response to vaccination Ann Trop Med Parasitol 1985 79 6 563 573 3834841 10.1080/00034983.1985.11811963

[B16] Usen S Milligan P Ethevenaux C Greenwood B Mulholland K Effect of fever on the serum antibody response of Gambian children to Haemophilus influenzae type b conjugate vaccine Pediatr Infect Dis J 2000 19 5 444 449 10.1097/00006454-200005000-00010 10819341

[B17] Cunnington A Riley E Suppression of vaccine responses by malaria: insignificant or overlooked? Expert Rev Vaccines 2010 9 4 409 429 10.1586/erv.10.16 20370551

[B18] Wilson ME Fineberg HV Colditz GA Geographic latitude and the efficacy of bacillus Calmette-Guérin vaccine Clin Infect Dis 1995 20 4 982 991 10.1093/clinids/20.4.982 7795103

[B19] Newport M Huxley C Huston S Hawrylowicz C Oostra B Williamson R Levin M A mutation in the interferon-gamma-receptor gene and susceptibility to mycobacterial infection N Engl J Med 1996 335 26 1941 1949 10.1056/NEJM199612263352602 8960473

[B20] Dorman S Holland S Mutation in the signal-transducing chain of the interferon-γ receptor and susceptibility to mycobacterial infection J Clin Invest 1998 101 11 2364 2369 10.1172/JCI2901 9616207 PMC508825

[B21] Kampmann B Hemingway C Stephens A Davidson R Goodsall A Anderson S Nicol M Schölvinck E Relman D Waddell S Acquired predisposition to mycobacterial disease due to autoantibodies to IFN-γ J Clin Invest 2005 115 9 2480 2488 10.1172/JCI19316 16127458 PMC1190367

[B22] Black G Weir R Floyd S Bliss L Warndorff D Crampin A Ngwira B Sichali L Nazareth B Blackwell J BCG-induced increase in interferon-gamma response to mycobacterial antigens and efficacy of BCG vaccination in Malawi and the UK: two randomised controlled studies The Lancet 2002 359 9315 1393 1401 10.1016/S0140-6736(02)08353-8 11978337

[B23] Lalor M Ben-Smith A Gorak-Stolinska P Weir R Floyd S Blitz R Mvula H Newport M Branson K McGrath N Population differences in immune responses to Bacille Calmette-Guérin vaccination in infancy J Infect Dis 2009 199 6 795 800 10.1086/597069 19434928 PMC3276835

[B24] Miles D van der Sande M Kaye S Crozier S Ojuola O Sanneh M Touray E Waight P Rowland-Jones S Whittle H CD4^+ ^T cell responses to cytomegalovirus in early life: a prospective birth cohort study J Infect Dis 2008 197 5 658 662 10.1086/527418 18279047

[B25] Walther B Miles D Crozier S Waight P Palmero M Ojuola O Touray E van der Sande M Whittle H Rowland-Jones S Placental malaria is associated with reduced early life weight development of affected children independent of low birth weight Malar J 2010 9 1 16 10.1186/1475-2875-9-16 20074331 PMC2841609

[B26] Hayes K Alford C Britt W Antibody response to virus-encoded proteins after cytomegalovirus mononucleosis J Infect Dis 1987 156 4 615 621 10.1093/infdis/156.4.615 3040869

[B27] Ismail M Ordi J Menendez C Ventura P Aponte J Kahigwa E Hirt R Cardesa A Alonso P Placental pathology in malaria: a histological, immunohistochemical, and quantitative study Hum Pathol 2000 31 1 85 93 10.1016/S0046-8177(00)80203-8 10665918

[B28] Kaye S Miles D Antoine P Burny W Ojuola O Kaye P Rowland-Jones S Whittle H van der Sande M Marchant A Virological and immunological correlates of mother to child transmission of cytomegalovirus in The Gambia J Infect Dis 2008 197 9 1307 1314 10.1086/586715 18422443

[B29] Pass RF Epidemiology and transmission of cytomegalovirus J Infect Dis 1985 152 2 243 248 10.1093/infdis/152.2.243 2993429

[B30] Njie R Bell A Jia H Croom-Carter D Chaganti S Hislop A Whittle H Rickinson A The effects of acute malaria on Epstein-Barr virus (EBV) load and EBV-specific T cell immunity in Gambian children J Infect Dis 2009 199 1 31 38 10.1086/594373 19032105

[B31] Moormann A Chelimo K Sumba O Lutzke M Ploutz-Snyder R Newton D Kazura J Rochford R Exposure to holoendemic malaria results in elevated Epstein-Barr virus loads in children J Infect Dis 2005 191 8 1233 1238 10.1086/428910 15776368

[B32] Moormann A Chelimo K Sumba P Tisch D Rochford R Kazura J Exposure to holoendemic malaria results in suppression of Epstein-Barr virus-specific T cell immunosurveillance in Kenyan children J Infect Dis 2007 195 6 799 808 10.1086/511984 17299709

[B33] Miles D van der Sande M Crozier S Ojuola O Palmero M Sanneh M Touray E Rowland-Jones S Whittle H Ota M Effect of Antenatal and Postnatal environment on CD4 T-cell Responses to BCG in healthy Gambian infants Clin Vaccine Immunol 2008 15 6 995 1002 10.1128/CVI.00037-08 18400973 PMC2446611

[B34] Moore S Jalil F Ashraf R Szu S Prentice A Hanson L Birth weight predicts response to vaccination in adults born in an urban slum in Lahore, Pakistan Am J Clin Nutr 2004 80 2 453 459 15277170 10.1093/ajcn/80.2.453

[B35] McDade T Beck M Kuzawa C Adair L Prenatal undernutrition, postnatal environments, and antibody response to vaccination in adolescence Am J Clin Nutr 2001 74 4 543 548 11566655 10.1093/ajcn/74.4.543

[B36] van der Sande M Kaye S Miles D Waight P Jeffries D Ojuola O Palmero M Pinder M Ismaili J Flanagan K Risk factors for and clinical outcome of congenital cytomegalovirus infection in a peri-urban west-African birth cohort PLoS ONE 2007 2 6 e492 10.1371/journal.pone.0000492 17551573 PMC1876257

[B37] Schim van der Loeff M Sarge-Njie R Ceesay S Awasana A Jaye P Sam O Jaiteh K Cubitt D Milligan P Whittle H Regional differences in HIV trends in The Gambia: results from sentinel surveillance among pregnant women AIDS 2003 17 12 1841 1846 10.1097/00002030-200308150-00014 12891071

[B38] Malhotra I Mungai P Wamachi A Kioko J Ouma J Kazura J King C Helminth- and Bacillus Calmette-Guérin-induced immunity in children sensitized in utero to filariasis and schistosomiasis J Immunol 1999 162 11 6843 6848 10352306

[B39] Wammes L Hamid F Wiria A de Gier B Sartono E Maizels R Luty A Fillié Y Brice G Supali T Regulatory T cells in human geohelminth infection suppress immune responses to BCG and *Plasmodium falciparum *Eur J Immunol 2010 40 2 437 442 10.1002/eji.200939699 20063313

[B40] Elias D Britton S Aseffa A Engers H Akuffo H Poor immunogenicity of BCG in helminth infected population is associated with increased in vitro TGF-β production Vaccine 2008 26 31 3897 3902 10.1016/j.vaccine.2008.04.083 18554755

[B41] LaBeaud A Malhotra I King M King C King C Do antenatal parasite infections devalue childhood vaccination? PLoS Negl Trop Dis 2009 3 5 e442 10.1371/journal.pntd.0000442 19478847 PMC2682196

[B42] Schwarz N Adegnika A Breitling L Gabor J Agnandji S Newman R Lell B Issifou S Yazdanbakhsh M Luty A Placental malaria increases malaria risk in the first 30 months of life Clin Infect Dis 2008 47 8 1017 1025 10.1086/591968 18781874

[B43] Urban B Ferguson D Pain A Willcox N Plebanski M Austyn J Roberts D *Plasmodium falciparum*-infected erythrocytes modulate the maturation of dendritic cells Nature 1999 400 6739 73 77 10.1038/21900 10403251

[B44] Ho M Webster H Green B Looareesuwan S Kongchareon S White N Defective production of and response to IL-2 in acute human falciparum malaria J Immunol 1988 141 8 2755 2759 2971729

